# Characteristics of hemolytic uremic syndrome in patients from a pediatric hospital in Peru, 2010-2020

**DOI:** 10.17843/rpmesp.2023.402.12708

**Published:** 2023-06-26

**Authors:** Lisbeth Varenia Carrasco-Oros, Noé Atamari-Anahui, Alcida Goñi-Fano, Claudia Sosa-Carmelo, Eduardo Jesús Guzmán-Quispe, Nadin Conto-Palomino, Basem Rodolfo Cabrera-Villacriz, Carla Lisette Apeña-Cabrera

**Affiliations:** 1 Instituto Nacional de Salud del Niño-Breña, Lima, Peru. Instituto Nacional de Salud del Niño-Breña Lima Peru; 2 Faculty of Human Medicine, San Marcos National University., Lima, Peru. Universidad Nacional Mayor de San Marcos Faculty of Human Medicine San Marcos National University Lima Peru; 3 Research Unit for the Generation and Synthesis of Health Evidence, Vice Rectorate for Research, San Ignacio de Loyola University, Lima, Peru. San Ignacio de Loyola University Research Unit for the Generation and Synthesis of Health Evidence Vice Rectorate for Research San Ignacio de Loyola University Lima Peru; 4 Pediatrics Service, Nacional Arzobispo Loayza Hospital, Lima, Peru. Pediatrics Service Nacional Arzobispo Loayza Hospital Lima Peru

**Keywords:** Hemolytic uremic syndrome, Renal Replacement Therapy, Child, Peru

## Abstract

This study aimed to describe the clinical-epidemiological, laboratory, treatment, and follow-up characteristics of patients with hemolytic uremic syndrome (HUS). The medical records of patients with HUS hospitalized at the Instituto Nacional de Salud del Niño-Breña (INSN-B) (Lima, Peru) were reviewed. We evaluated 83 patients. The median age was 22 months (interquartile range: 14 to 30 months). Of the sample, 71.1% (59) registered previous use of antibiotics. Seventy-two (86.8%) had oligoanuria and 62 (74.6%) had diarrhea. Five cultures were positive (two enterohaemorrhagic Escherichia coli). Forty-nine (59%) required renal replacement therapy. No patient died during hospitalization. At one year of follow-up, seven patients developed post-HUS nephropathy. In conclusion, in INSN-B, the median age was like previous years and there was a higher frequency of oligoanuria, and renal replacement therapy compared to previous reports.

## INTRODUCTION

Hemolytic uremic syndrome (HUS) is a condition characterized by non-immune microangiopathic hemolytic anemia, thrombocytopenia and acute kidney injury ^1^. Infection-associated HUS is most frequently caused by enterohemorrhagic *Escherichia coli* (EHEC) producing Shiga toxin (STx) and is the most common cause in children under five years of age^1^.

Argentina is the Latin-American country with the highest incidence of HUS with 5.95 cases/100,000 children under five years of age ^2^, followed by Chile with 3.4 cases/100,000 children under five years of age ^3^. In Peru, the incidence of HUS has increased from 0.55 (1982-1986) to 2.02 cases/1000 hospitalized pediatric patients (2002-2006) ^4^^,^^5^. 

The study of HUS is important because this condition can lead to persistent and non-reversible kidney damage ^1^^,^^6^. In addition, HUS can be prevented ^7^ because it is an infectious syndrome due to the consumption of contaminated food, which is why it is actively surveilled in some countries ^2^^,^^3^. 

In Peru, there is limited information describing the epidemiological and clinical characteristics of HUS in recent years ^4^^,^^5^. For this reason, this study aimed to describe the clinical, epidemiological and laboratory characteristics, treatment and follow-up of patients with HUS in a hospital in Lima, Peru, between 2010 and 2020.

**KEY MESSAGES**

Motivation for the study: There are few studies in Peru on hemolytic uremic syndrome.

Main findings: Between the years 2010 to 2020, the age at diagnosis has not changed; however, more patients presented oliguria and required more renal replacement therapy (peritoneal dialysis) compared to previous years.

Implications: This syndrome is an important cause of renal damage in children; therefore, its surveillance and notification are necessary. In addition, measures of prevention and early recognition of the disease must be implemented, since this condition is generally caused by consumption of contaminated food.

## THE STUDY

### Design and population

This was a descriptive study. Data was obtained from the medical records of hospitalized patients with HUS between January 2010 and December 2020, from the Instituto Nacional de Salud del Niño- Breña (INSN-B) in Lima, Peru.

We included medical records of patients under 18 years old, who were diagnosed with HUS (ICD 10 code: D59.3), defined by non-immune microangiopathic hemolytic anemia, thrombocytopenia and impaired renal function ^1^^,^^8^. Medical records of patients who, prior to being diagnosed with HUS, had chronic kidney disease (CKD), proteinuria or microalbuminuria under study or treatment, renal replacement therapy (RRT) for any disease were excluded. Two medical records were excluded from the analysis (one because of CKD and the other because the file was missing).

### Study variables and procedures

We considered the following variables: age (categorized as <1 year, 1 to 2 years, 2 to 5 years, and >5 years), sex, origin (Lima and provinces), medical history, genetic cause, antibiotic use prior to admission, hypertension on admission, clinical manifestations on admission, time of illness and hospitalization, laboratory tests on admission, treatment (type of hydration, use of antibiotics, use of blood products and RRT), complications during hospitalization, admission to the intensive care unit (ICU) and follow-up (post-HUS nephropathy, hypertension and proteinuria at one year after discharge). The information was obtained from the physical medical records (archive area) after approval and authorization from the medical records area of the hospital.

### Statistical analysis

The information was collected in a Microsoft Excel ® (Windows 10 version) database. This database was reviewed independently by two researchers (NAA and NCP). The analysis was performed in STATA version 16 (StataCorp LP, College Station, Texas, United States). Qualitative variables were described with absolute and relative frequencies, and quantitative variables were described using medians and interquartile ranges (IQR), after evaluation of the assumption of normality with the Shapiro-Wilk test.

### Ethical aspects

The study was approved by the INSN-B research ethics committee (Document No. 010-2022-CIEI-INSN). Data confidentiality was maintained by using a numerical code to avoid identification of the participants. Informed consent was not requested because the information was collected directly from the medical records.

## FINDINGS

A total of 83 participants were included in the study. The median age was 22 months (IQR: 14-30) and 59.1% (n=49) were younger than two years (Table 1). Fifty-three percent (n=44) were female and 65.1% (n=54) were from Lima. Three patients had a medical history (two were patients with Down syndrome and one had patent ductus arteriosus). None had a history of familial HUS. A diacylglycerol kinase epsilon gene mutation caused HUS in one of the patients. At admission, 71.1% (n=59) reported antibiotic use and 50.6% (n=42) had hypertension.

**Table 1:** Clinical and epidemiological characteristics of patients with hemolytic uremic syndrome.

Characteristics	Total n=83 (%)
Age (months) ^a^	22 (14-30)
< 1 year	12 (14.5)
1 to 2 years	37 (44.6)
2 to 5 years	31 (37.3)
> 5 years	3 (3.6)
Sex	
Female	44 (53.0)
Male	39 (47.0)
Origin	
Provinces	29 (34.9)
Lima	54 (65.1)
Antibiotic use prior to admission	
No	24 (28.9)
Yes	59 (71.1)
Hypertension on admission	
No	41 (49.4)
Yes	42 (50.6)
Clinical manifestations on admission ^b^	
Oliguria	72 (86.8)
Pallor	69 (83.1)
Edema	63 (75.9)
Diarrhea	62 (74.6)
Vomiting	57 (68.7)
Dehydration	34 (40.9)
Neurological symptoms	
Irritability	22 (26.5)
Drowsiness	14 (16.9)
Convulsions	13 (15.6)
Time of illness (days)^a^	6 (5-7)
Time of hospitalization (days)^a^	11 (8-16)

a Median and interquartile range, ^b^ may have more than one clinical manifestation.

Oliguria was the most frequent clinical manifestation on admission with a median of 2 days (IQR: 2-3). Dehydration was reported in 34 (40.9%) patients (5 mild, 28 moderate and 1 severe). Sixty-two (74.6%) patients had diarrhea and thirteen (15.6%) reported seizures prior to admission. The median time of illness was six days (IQR: 5-7) and the time of hospitalization was 11 days (IQR: 8-16). Seventy-two (86.7%) patients had acute kidney injury on admission, 65/72 (90.3%) had oliguria and 29/72 (40.3%) some degree of dehydration.

The median hemoglobin was 6.7 g/dL (IQR: 5.4-8.5) and 44 (53%) patients had severe anemia (hemoglobin <7 g/dL). Twenty-five (30.1%) had platelets <50,000 cells/mm^3^. Median serum sodium was 133 mmol/L (IQR: 129-137) and 29 (34.9%) patients had sodium <130 mmol/L. We also found elevated lactate dehydrogenase with a median of 4693 U/L (IQR: 3320-6053) and decreased C3 (median: 85.6 mg/dL; IQR: 69.0-103.0) and bicarbonate (median: 12.6 mEq/L; IQR: 10.1-17.5) on admission (Table 2). All patients presented an increase of the spot urine protein/creatinine ratio. Of the 51 stool culture samples, five were positive (two for enterohemorrhagic *Escherichia coli*, two for enteropathogenic *Escherichia coli* and one for *Clostridium difficile*).

**Table 2:** Laboratory characteristics of patients with hemolytic uremic syndrome.

Laboratory results ^a^	Total (n=83)
Hemoglobin (g/dL)	6,7 (5,4-8,5)
Leucocytes (cel/mm^3^)	11980 (9960-17820)
Platelets (cel/mm^3^)	70000 (40000-103000)
Reticulocytes (%)	3,8 (2,3-5,7)
C reactive protein (mg/dL)	1,6 (0,5-2,3)
Albumin (g/dL)	3,2 (3,0-3,5)
Sodium (mmol/L)	133 (129-137)
Potassium (mmol/L)	4 (3,5-4,8)
Lactate dehydrogenase (U/L)	4693 (3320-6053)
Urea (mg/dL)	179 (108-245)
Creatinine (mg/dL)	4,0 (2,1-5,7)
Amylase (mg/dL)	43 (29,5-79,5)
Lipase (mg/dL)	70 (43,0-121,9)
C3 mg/dL	85,6 (69,0-103,0)
C4 mg/dL	23 (16-29)
Calcium mg/dL	8,7 (8,2-9,4)
Uric acid mg/dL	12,6 (8,9-16,2)
pH	7,38 (7,31-7,44)
HCO_3_ (mEq/L)	12,6 (10,1-17,5)
Protein/creatinine	5 (2,6-8,5)

a Median and interquartile range.

During hospitalization, 49 patients (59%) required RRT and the most frequent complication was hypertensive crisis followed by seizures (Table 3). Eight patients were admitted to the intensive care unit (three had hypertensive crisis, two had metabolic disorder, and one had myocardial damage, pancreatitis and pneumonia).Also, one patient had leukemoid reaction, one had ataxia and metabolic encephalopathy, one had pneumonia, and two had abdominal distension. No patient died during hospitalization.

**Table 3:** Treatment characteristics and complications of patients with hemolytic uremic syndrome.

Treatment	Total (n=83)
Basal hydration at admission	17 (20.5)
Water restriction at admission	66 (79.5)
Antibiotics during hospitalization	36 (43.4
Use of blood products	
Red blood cell units	76 (91.6)
Platelets	30 (36.1)
Fresh frozen plasma	4 (4.8)
Renal replacement therapy	
Peritoneal dialysis	48 (57.8)
Hemodialysis	1 (1.2)
Complications during hospitalization ^a^	35 (42.2)
Hypertensive crisis	20 (24.1)
Convulsions	10 (12.0)
Peritoneal dialysis -related peritonitis	9 (10.8)
Sepsis	6 (7.2)
Rectal prolapse	3 (3.6)
Pancreatic involvement	2 (2.4)
Intussusception	2 (2.4)
Hemorrhagic colitis	1 (1.2)
Myocardial damage	1 (1.2)
Admission to ICU	8 (9.6)

ICU: intensive care unit.

a They may have more than one complication.

After hospitalization, 72 (86.7%) patients had at least one follow-up appointment in the nephrology service. The median follow-up time was 1.3 years (IQR: 0.4-4.5). Twenty-seven (37.5%) patients had a follow-up time longer than one year. Seven patients had post-HUS nephropathy, five had hypertension and eight had proteinuria at one year of follow-up.

## DISCUSSION

In our study, the median age of patients with HUS was similar to what has been previously reported (Peru, 18 months) ^5^, (Chile 22.8 months) ^6^, (Portugal and Norway, 24 months) ^9^^,^^10^. Infection-associated HUS occurs more frequently in children and is caused by a higher glomerular expression of the STx receptor Globotriaosylceramide 3, unlike in adults who have anti-STx antibodies and thus avoid the disease ^1^.

Antibiotics were used prior to admission in 71.1% of the patients, which is lower than what has been reported in previous years in Peru (90%) ^5^, and higher than in Chile (52%) ^6^. Antibiotics such as β-lactams and trimethoprim/sulfamethoxazole increase the risk of HUS by inducing the expression and release of STx ^11^, although the evidence is still conflicting, their use is not recommended in suspected or confirmed cases of STx-producing HUS ^7^^,^^12^.

Oliguria at admission was found in 86.8% of the patients, which is higher than what has been reported by other studies ^9^^,^^10^. Oliguria is caused by renal endothelial injury, which produces microvascular thrombi and later intrinsic acute renal injury ^8^. It frequently occurs in HUS associated with diarrhea ^10^ and increases the risk of chronic renal damage ^6^. In the study, 29 patients presented dehydration and acute kidney injury. Dehydration is associated with the need for RRT and with mortality ^13^; therefore, adequate hydration is important in those patients who do not have signs of water overload regardless of renal function ^14^.

Diarrhea prodrome was found in 74.6% of the patients, which is less than that previously reported ^5^^,^^6^^,^^10^. This symptom occurs in around 90% of the cases between day 3 to 8 after ingestion of contaminated food such as semi-cooked meats, unpasteurized dairy products, as well as contaminated water and vegetables ^1^^,^^7^.

Thirteen (15.6%) patients had seizures before admission, this figure is lower than that reported by previous Peruvian studies with 33% (1976-2002) ^4^ and 28% (2002-2009) ^5^. The frequency of this symptom is estimated to range from 10 to 25% ^15^, in addition, it increases the risk of renal sequelae and mortality ^6^. Neurological manifestations such as seizures can be caused by the alteration of neuronal cells and by the release of cytokines produced by STx ^1^^,^^8^, and the decrease in their frequency may be due to early diagnosis and optimal management in recent years.

All patients presented alterations in the laboratory test results, which is similar to other reports ^3^^,^^5^^,^^10^. These alterations included anemia, increased lactate dehydrogenase, thrombocytopenia and acute renal injury, all of which are typical of endothelial injury and oxidative stress ^1^. Fifty-three percent of the participants had severe anemia, which is less than that reported between 2002-2009 (65%) ^5^. We also found a decrease in C3. This immunological marker has recently been studied in HUS cases and it decreases due to the activation and consumption of the complement pathway ^1^; some series report that it is associated with greater neurological involvement and RRT ^16^.

Five stool cultures were positive. A previous Peruvian study reported that enteropathogenic *Escherichia coli* was the most frequent one (5/12 positive samples) ^5^; our findings also showed EHEC, which is associated with HUS ^1^. The low frequency may be due to the high antibiotic use prior to admission, which is similar to what was found by other reports ^6^, in addition to the absence of an established protocol for the study of EHEC and the lack of specific cultures to detect the bacteria ^17^.

Forty-nine (59%) patients required RRT, which is higher than that found by studies from Peru (46%) ^5^ and Chile (42.4%) ^6^. Of these patients, 47/49 (95.9%) had oliguria and 41/49 (83.7%) had water restriction on admission. Both characteristics are associated with RRT ^14^, which could explain their high frequency. In addition, this type of treatment is not available in all hospitals in Peru, so it is expected that patients with some degree of severity or possible complications requiring dialysis would be referred to INSN-B. RRT is used only in cases of patients with anuria for more than 24 hours, refractory hydroelectrolytic disorders and hypervolemia ^18^ and its use ranges from 30% to 60% of the cases ^1^^,^^8^. Peritoneal dialysis is the most commonly used type of RRT in INSN-B, which is similar to hospitals in Chile ^6^ and Argentina ^18^.

Complications during hospitalization were similar to those described in other studies ^6^^,^^10^, with a lower frequency of hypertensive crises and convulsions compared to a previous Peruvian study ^5^. Peritoneal dialysis catheter-associated peritonitis continues to be an important complication, as reported by other studies ^6^^,^^18^, and therefore experience, management and care are necessary to reduce its frequency.

Seven patients presented post-HUS nephropathy during follow-up. In another Peruvian hospital, post-renal sequelae were reported in 7/12 patients who were followed up for more than six months ^20^, with proteinuria and hypertension being the most frequent manifestations, which is similar to our findings. Anuria and the need for RRT have been associated with chronic renal damage ^6^^,^^19^; these characteristics were frequent in our study. No patient died during hospitalization. A mortality rate of 2.3% was reported inPeru between 2002-2009 ^5^; it was 2.9% in Chile between 1990-2003 ^6^. Mortality is expected to decrease in recent years ^20^ due to access to RRT and optimal management of complications.

One of the limitations of our study is that we included data from a single hospital, so the results cannot be generalized to the national level. Likewise, serotyping of positive stool samples could not be performed. Nevertheless, our results show the current characteristics of patients with HUS from a national referral hospital in Peru.

In conclusion, the median age of patients with HUS in INSN-B was similar to previous reports, and half of the patients used antibiotics before admission. Oliguria was the most frequent clinical manifestation, and the most commonly used RRT was peritoneal dialysis. No patient died during hospitalization. Since HUS is associated with contaminated food, we recommend its epidemiological surveillance, similar to other countries. In addition, HUS isolation techniques should be implemented in the microbiology laboratories of the national referral centers of the Ministry of Health.
