# Supplementary material for: Characteristics of hemolytic uremic syndrome in patients from a pediatric hospital in Peru, 2010-2020
Source: Rev Peru Med Exp Salud Publica. 2023 Jun 26;40(2):207–12. doi: 10.17843/rpmesp.2023.402.12708 (PMC11060181; doi:10.17843/rpmesp.2023.402.12708)
Supplement: Translation

# Translation: Características del síndrome urémico hemolítico en pacientes de un hospital pediátrico de Perú, 2010-2020

**Authors:** Lisbeth Varenia Carrasco-Oros, Noé Atamari-Anahui, Alcida Goñi-Fano, Claudia Sosa-Carmelo, Eduardo Jesús Guzmán-Quispe, Nadin Conto-Palomino, Basem Rodolfo Cabrera-Villacriz, Carla Lisette Apeña-Cabrera

**Affiliations:** 1 Instituto Nacional de Salud del Niño-Breña, Lima, Perú.; 2 Facultad de Medicina Humana, Universidad Nacional Mayor de San Marcos, Lima, Perú.; 3 Unidad de Investigación para la Generación y Síntesis de Evidencias en Salud, Vicerrectorado de Investigación, Universidad San Ignacio de Loyola, Lima, Perú.; 4 Servicio de pediatría, Hospital Nacional Arzobispo Loayza, Lima, Perú.

**Keywords:** Síndrome Hemolítico-Urémico, Terapia de Reemplazo Renal, Niños, Perú

## Abstract

El propósito del presente estudio fue describir las características clínicas, epidemiológicas, laboratoriales, tratamiento y seguimiento de pacientes con síndrome urémico hemolítico (SUH). Se revisaron las historias clínicas de los pacientes con SUH hospitalizados en el Instituto Nacional de Salud del Niño-Breña (INSN-B) de Lima, Perú. Se incluyeron a 83 pacientes. La mediana de edad fue de 22 meses. El 71,1% (n=59) registró uso previo de antibióticos. El 86,8% (n=72) tuvieron oligoanuria y el 74,6% (n=62) diarrea. Cinco cultivos fueron positivos (dos *Escherichia coli* enterohemorrágica). Cuarenta y nueve (59%) requirieron terapia de reemplazo renal. Ningún paciente falleció durante la hospitalización. Al año del seguimiento, siete pacientes presentaron nefropatía pos-SUH. En conclusión, en el INSN-B, la mediana de edad fue similar que años anteriores y hubo una mayor frecuencia de oligoanuria y terapia de reemplazo renal en comparación con reportes previos.

## INTRODUCCIÓN

El síndrome urémico hemolítico (SUH) se caracteriza por anemia hemolítica microangiopática no inmune, trombocitopenia y lesión renal aguda ^1^. El SUH asociado a infección es el más frecuente producido por *Escherichia coli* enterohemorrágica (ECEH) productora de toxina Shiga (STx) y es la causa más común en menores de cinco años ^1^.

En Latinoamérica, Argentina es el país que mayor incidencia de SUH con 5,95 casos/100 000 niños menores de cinco años ^2^, seguido de Chile con 3,4 casos/100 000 menores de cinco años ^3^. En Perú, la incidencia de SUH se ha incrementado de 0,55 (periodo 1982-1986) a 2,02 casos/1000 pacientes pediátricos hospitalizados (periodo 2002-2006) ^4^^,^^5^.

El estudio del SUH es importante porque puede generar daño renal persistente y no reversible ^1^^,^^6^; además, se puede prevenir ^7^ por ser un síndrome infeccioso por consumo de alimentos contaminados, por lo que es motivo de vigilancia activa en algunos países ^2^^,^^3^.

En el Perú, existe limitada información sobre el SUH ^4^^,^^5^, que describa el comportamiento epidemiológico y clínico en los últimos años. Por esta razón, el objetivo del presente trabajo fue describir las características clínicas, epidemiológicas, laboratoriales, tratamiento y seguimiento de pacientes con SUH en un hospital de Lima, Perú, entre el 2010 al 2020.

**MENSAJES CLAVE**

Motivación para realizar el estudio: Existen limitados estudios actuales en Perú sobre el síndrome urémico hemolítico.

Principales hallazgos: Entre los años 2010 al 2020, la edad de diagnóstico no ha cambiado; sin embargo, los pacientes presentaron mayor oligoanuria y requirieron más terapia de reemplazo renal (diálisis peritoneal) a comparación de años previos.

Implicancias: Este síndrome es una importante causa de daño renal en los niños, siendo necesaria su vigilancia y notificación; además, de implementar medidas de prevención y reconocimiento temprano de la enfermedad, al ser producida generalmente por consumo de alimentos contaminados.

## EL ESTUDIO

### Diseño y población

Se realizó un estudio descriptivo. Los datos procedieron de las historias clínicas de los pacientes hospitalizados con diagnóstico de SUH entre enero del 2010 a diciembre del 2020, en el Instituto Nacional de Salud del Niño-Breña (INSN-B) de Lima, Perú.

Se incluyeron historias clínicas con registro de pacientes menores de 18 años con diagnóstico de SUH (código CIE 10: D59.3) definido por anemia hemolítica microangiopática no inmune, trombocitopenia y deterioro de la función renal ^1^^,^^8^. Se excluyeron historias clínicas de pacientes con enfermedad renal crónica (ERC), proteinuria o microalbuminuria en estudio o tratamiento, terapia de reemplazo renal (TRR) por alguna enfermedad, todos estos criterios antes del diagnóstico de SUH. Dos historias clínicas fueron excluidas del análisis (uno por ERC y otro porque no se ubicó la historia clínica en el archivo).

### Variables de estudio y procedimientos

Se consideraron a las variables como la edad (categorizada en <1 año, 1 a 2 años, 2 a 5 años y >5 años), sexo, procedencia (Lima y provincias), antecedentes médicos, causa genética, uso de antibióticos previo al ingreso, hipertensión al ingreso, manifestaciones clínicas al ingreso, tiempo de enfermedad y hospitalización, exámenes de laboratorio al ingreso, tratamiento (tipo de hidratación, uso de antibióticos, uso de hemoderivados y TRR), complicaciones durante la hospitalización, ingreso a la unidad de cuidados intensivos y seguimiento (desarrollo de nefropatía pos-SUH, hipertensión y proteinuria al año). La información fue obtenida de las historias clínicas físicas (área de archivo) posterior a la aprobación del estudio y autorización del área de registros médicos del hospital.

### Análisis estadístico

Se diseñó una base de datos en el programa Microsoft Excel ® (versión para Windows 10) para recopilar la información. Esta base fue revisada por dos investigadores (NAA y NCP) de manera independiente. El análisis se realizó en el programa STATA versión 16 (StataCorp LP, College Station, Texas, Estados Unidos). Se describieron las variables cualitativas mediante frecuencias absolutas y relativas, y las variables cuantitativas mediante medianas y rangos intercuartílicos (RIC), previa evaluación del supuesto de normalidad, con la prueba de Shapiro-Wilk.

### Aspectos éticos

El estudio fue aprobado por el comité de ética en investigación del INSN-B (Oficio N.° 010-2022-CIEI-INSN). Se respetó la confidencialidad de los datos usando un código numérico para evitar la identificación de los participantes. No se solicitó consentimiento informado porque la información se recolectó directamente de las historias clínicas.

## HALLAZGOS

En el periodo de estudio se incluyeron 83 participantes. La mediana de edad fue 22 meses (RIC: 14-30). El 59,1% (n=49) era menor de dos años (Tabla 1). El 53% (n=44) eran mujeres y el 65,1% (n=54) procedían de Lima. Tres pacientes tenían antecedentes médicos (dos con síndrome de Down y uno con persistencia del conducto arterioso). Ninguno tenía antecedente de SUH familiar. Un paciente tuvo como causa genética de SUH a la mutación del gen diacilglicerol quinasa épsilon. El 71,1% (n=59) al ingreso reportó uso de antibióticos y el 50,6% (n=42) tenía hipertensión.

**Tabla 1:** Características clínicas y epidemiológicas de los pacientes con síndrome urémico hemolítico.

Características	Total n=83 (%)
Edad (meses) ^a^	22 (14-30)
< 1 año	12 (14,5)
1 a 2 años	37 (44,6)
2 a 5 años	31 (37,3)
> 5 años	3 (3,6)
Sexo	
Femenino	44 (53,0)
Masculino	39 (47,0)
Procedencia	
Provincias	29 (34,9)
Lima	54 (65,1)
Uso de antibióticos previo al ingreso	
No	24 (28,9)
Sí	59 (71,1)
Hipertensión al ingreso	
No	41 (49,4)
Sí	42 (50,6)
Manifestaciones clínicas al ingreso ^b^	
Oligoanuria	72 (86,8)
Palidez	69 (83,1)
Edema	63 (75,9)
Diarrea	62 (74,6)
Vómitos	57 (68,7)
Deshidratación	34 (40,9)
Síntomas neurológicos	
Irritabilidad	22 (26,5)
Somnolencia	14 (16,9)
Convulsiones	13 (15,6)
Tiempo de enfermedad (días)^a^	6 (5-7)
Tiempo de hospitalización (días)^a^	11 (8-16)

a Mediana y rango intercuartílico, ^b^ pueden tener más de una manifestación clínica.

La oligoanuria fue la manifestación clínica más frecuente al ingreso con una mediana de 2 días (RIC: 2-3).La deshidratación fue reportada en 34 (40,9%) pacientes (5 leves, 28 moderados y 1 severo). Sesenta y dos (74,6%) tuvieron diarrea y trece (15,6%) pacientes reportaron convulsiones antes del ingreso. La mediana del tiempo de enfermedad fue de seis días (RIC: 5-7) el tiempo de hospitalización fue de 11 días (RIC: 8-16). Setenta y dos (86,7%) pacientes tuvieron lesión renal aguda al ingreso, 65/72 (90,3%) tenían oligoanuria y 29/72 (40,3%) algún grado de deshidratación.

La mediana de hemoglobina fue de 6,7 g/dL (RIC: 5,4-8,5) y 44 (53%) pacientes tuvieron anemia severa (hemoglobina <7 g/dL). Veinticinco (30,1%) tuvieron plaquetas <50 000 cel/mm^3^. La mediana del sodio sérico fue de 133 mmol/L (RIC: 129-137) y 29 (34,9%) tuvieron sodio < 130 mmol/L. También se encontró elevación del lactato deshidrogenasa con una mediana de 4693 U/L (RIC: 3320-6053) y disminución del C3 (mediana: 85,6 mg/dL; RIC: 69,0-103,0) y del bicarbonato (mediana: 12,6 mEq/L; RIC: 10,1-17,5) al ingreso (Tabla 2). Todos los pacientes tuvieron elevación una relación de proteína/creatinina en orina al azar. De las 51 muestras de coprocultivo reportadas, cinco fueron positivas (dos a *Escherichia coli* enterohemorrágica, dos a *Escherichia coli* enteropatogénica y uno a *Clostridium difficile*).

**Tabla 2:** Características laboratoriales de pacientes con síndrome urémico hemolítico.

Características laboratoriales ^a^	Total (n=83)
Hemoglobina (g/dL)	6,7 (5,4-8,5)
Leucocitos (cel/mm^3^)	11980 (9960-17820)
Plaquetas (cel/mm^3^)	70000 (40000-103000)
Reticulocitos (%)	3,8 (2,3-5,7)
Proteína c reactiva (mg/dL)	1,6 (0,5-2,3)
Albúmina (g/dL)	3,2 (3,0-3,5)
Sodio (mmol/L)	133 (129-137)
Potasio (mmol/L)	4 (3,5-4,8)
Lactato deshidrogenasa (U/L)	4693 (3320-6053)
Urea (mg/dL)	179 (108-245)
Creatinina (mg/dL)	4,0 (2,1-5,7)
Amilasa (mg/dL)	43 (29,5-79,5)
Lipasa (mg/dL)	70 (43,0-121,9)
C3 mg/dL	85,6 (69,0-103,0)
C4 mg/dL	23 (16-29)
Calcio mg/dL	8,7 (8,2-9,4)
Ácido úrico mg/dL	12,6 (8,9-16,2)
pH	7,38 (7,31-7,44)
HCO_3_ (mEq/L)	12,6 (10,1-17,5)
Proteína/creatinina	5 (2,6-8,5)

a Mediana y rango intercuartílico.

Durante la hospitalización, 49 pacientes (59%) requirieron TRR y la complicación más frecuente fue la crisis hipertensiva seguida de las convulsiones (Tabla 3). Ocho pacientes ingresaron a la unidad de cuidados intensivos (tres tenían crisis hipertensiva, dos trastorno metabólico, uno daño miocárdico, pancreatitis y neumonía, respectivamente). Así también, un paciente tuvo reacción leucemoide, uno ataxia y encefalopatía metabólica, uno neumonía, y dos tuvieron distensión abdominal. Ningún paciente falleció durante la hospitalización.

**Tabla 3:** Características del tratamiento y complicaciones de los pacientes con síndrome urémico hemolítico.

Tratamiento	Total (n=83)
Hidratación basal al ingreso	17 (20,5)
Restricción hídrica al ingreso	66 (79,5)
Antibióticos durante la hospitalización	36 (43,4)
Uso de hemoderivados	
Paquete globular	76 (91,6)
Plaquetas	30 (36,1)
Plasma fresco congelado	4 (4,8)
Terapia de reemplazo renal	
Diálisis peritoneal	48 (57,8)
Hemodiálisis	1 (1,2)
Complicaciones en la hospitalización ^a^	35 (42,2)
Crisis hipertensiva	20 (24,1)
Convulsiones	10 (12,0)
Peritonitis asociada a catéter de diálisis peritoneal	9 (10,8)
Sepsis	6 (7,2)
Prolapso rectal	3 (3,6)
Compromiso pancreático	2 (2,4)
Intususcepción	2 (2,4)
Colitis hemorrágica	1 (1,2)
Daño miocárdico	1 (1,2)
Ingreso a UCI	8 (9,6)

UCI: unidad de cuidados intensivos.

a Pueden tener más de una complicación.

Posterior a la hospitalización, 72 (86,7%) pacientes tuvieron al menos un control por nefrología. La mediana de seguimiento fue de 1,3 años (RIC 0,4-4,5). Veintisiete (37,5%) pacientes tuvieron seguimiento mayor a un año. Siete pacientes presentaron nefropatía pos-SUH, cinco hipertensión y ocho tuvieron proteinuria al año de seguimiento.

## DISCUSION

En nuestro estudio, la mediana de edad de los pacientes con SUH fue similar a reportes previos (Perú, 18 meses) ^5^, (Chile 22,8 meses) ^6^, (Portugal y Noruega, 24 meses) ^9^^,^^10^. El SUH asociado a infección se presenta con mayor frecuencia en niños y se produce por una mayor expresión glomerular del receptor de STx Globotriaosilceramida 3, a diferencia de los adultos que presentan anticuerpos anti-STx y así evitan la enfermedad ^1^.

El uso de antibióticos fue del 71,1% previo al ingreso, menor a lo reportado en años anteriores en Perú (90%) ^5^, y superior a Chile (52%) ^6^. Los antibióticos como los β-lactámicos y el trimetropin/sulfametoxasol aumentan el riesgo de SUH por inducir la expresión y liberación de STx ^11^, aunque la evidencia aún es controversial, no se recomienda usarlo en aquellos casos sospechosos o confirmados de ECEH productores de STx ^7^^,^^12^.

El 86,8% tuvo oligoanuria al ingreso, superior a otros reportes ^9^^,^^10^. La oligoanuria se presenta por lesión del endotelio renal los cuales producen trombos microvasculares y posteriormente lesión renal aguda intrínseca ^8^. Se presenta con mayor frecuencia en el SUH asociado a diarrea ^10^ e incrementa el riesgo de daño renal crónico ^6^. En el estudio se observó que 29 pacientes tenían deshidratación y lesión renal aguda. La deshidratación se asocia a necesidad de TRR y mortalidad ^13^, por eso, es importante una adecuada hidratación en aquellos pacientes que no tengan signos de sobrecarga hídrica independientemente de la función renal ^14^.

El 74,6% tuvo pródromo de diarrea, menor a lo reportado en otros estudios ^5^^,^^6^^,^^10^. Esta sintomatología se presenta alrededor del 90% entre el día 3 a 8 después de la ingesta de alimentos contaminados como carnes semicocidas, productos lácteos no pasteurizados, aguas y vegetales contaminados ^1^^,^^7^.

Trece (15,6%) pacientes tuvieron convulsiones antes del ingreso, esta cifra es menor a reportes peruanos previos con 33% (periodo 1976-2002) ^4^ y 28% (periodo 2002-2009) ^5^. Se estima una frecuencia de esta sintomatología del 10 al 25% ^15^, además de incrementar el riesgo de secuela renal y mortalidad ^6^. Las manifestaciones neurológicas como las convulsiones se pueden explicar por la alteración de las células neuronales y por la liberación de citoquinas producidas por la STx ^1^^,^^8^, y la disminución de su frecuencia puede explicar por un reconocimiento precoz de la enfermedad y óptimo manejo en los últimos años.

Todos los pacientes presentaron alteraciones laboratoriales similares a otras series ^3^^,^^5^^,^^10^ caracterizado por anemia, lactato deshidrogenasa elevada, trombocitopenia y lesión renal aguda, todos propios de la lesión endotelial y estrés oxidativo ^1^. El 53% tenía anemia severa menor a lo reportado entre el 2002-2009 (65%) ^5^. También se observó una disminución del C3. Este marcador inmunológico es recientemente motivo de estudio en SUH y su disminución resulta de la activación y consumo de la vía del complemento ^1^, y algunas series reportan que se asocia a mayor compromiso neurológico y TRR ^16^.

Cinco coprocultivos fueron positivos. Un estudio peruano previo identificó con mayor frecuencia (5/12 muestras positivas) a *Escherichia coli* enteropatogénica ^5^, en nuestro estudio se reportó adicionalmente a la ECEH, la cual está asociada al SUH ^1^. La baja frecuencia de positividad puede explicarse por un mayor uso de antibióticos previos al ingreso similar a otros reportes ^6^, adicionado a la ausencia de un protocolo de estudio de ECEH y falta de cultivos especiales para detectar a la bacteria ^17^.

Cuarenta y nueve (59%) necesitaron TRR, superior a otras series (Perú, 46%) ^5^ y (Chile, 42,4%) ^6^. De estos pacientes, 47/49 (95,9%) tuvieron oligoanuria y 41/49 (83,7%) tuvieron restricción hídrica al ingreso. Ambas características se asocian a TRR ^14^, lo cual podría explicar su elevada frecuencia. Además, este tipo de tratamiento no está disponible en todos los centros hospitalarios de Perú, por lo que es esperable que los pacientes con algún grado de severidad o posible complicación que requiera diálisis sean referidos a nuestra institución. La TRR está reservada para pacientes con anuria más de 24 horas, trastornos hidroelectrolíticos refractarios e hipervolemia ^18^ y su uso oscila entre el 30 a 60% ^1^^,^^8^. En nuestra institución, el tipo de TRR más usado fue la diálisis peritoneal, similar a reportes en hospitales de Chile ^6^ y Argentina ^18^.

Las complicaciones en la hospitalización fueron similares a lo descrito en otras series ^6^^,^^10^, con una menor frecuencia de crisis hipertensivas y convulsiones respecto a un estudio peruano previo ^(^^5^. La peritonitis asociada a catéter de diálisis peritoneal sigue siendo una complicación importante, según lo reportado por otros centros ^6^^,^^18^, por lo que la experiencia, manejo y cuidados son necesarios para disminuir su frecuencia.

En el seguimiento, siete pacientes presentaron nefropatía pos-SUH. En otro hospital peruano se reportó una secuela posrrenal en 7/12 pacientes que tuvieron seguimiento mayor a seis meses ^20^, siendo la proteinuria e hipertensión las manifestaciones mas frecuentes, similar a nuestro estudio. La anuria y necesidad de TRR se asocia a daño renal crónico ^6^^,^^19^, y estas características fueron frecuentes en el estudio. Ningún paciente falleció durante la hospitalización. Entre el 2002-2009, se reportó una letalidad del 2,3% en Perú ^5^, y en Chile del 2,9% (1990-2003) ^6^, en los últimos años es esperable un descenso de la mortalidad ^20^, por el acceso a la TRR y al óptimo manejo de las complicaciones.

Dentro de las limitaciones podemos mencionar que este reporte es de un solo hospital, por lo que los resultados no se pueden generalizar al ámbito nacional. Asimismo, no se pudo realizar la serotipificación de las muestras positivas de heces. A pesar de ello, nuestros resultados muestran las características actuales de pacientes con SUH de un hospital de referencia nacional del Perú.

En conclusión, en el INSN-B la mediana de edad de los pacientes con SUH fue similar a reportes previos y la mitad recibió antibióticos antes del ingreso. La oligoanuria fue la manifestación clínica más frecuente y la modalidad de TRR más usada fue la diálisis peritoneal. Ningún paciente falleció durante la hospitalización. Se recomienda la vigilancia epidemiológica del SUH por estar asociado a alimentos contaminados, tal como se realiza en otros países. Además, se debe implementar las técnicas de aislamiento de ECEH en los laboratorios de microbiología en los centros de referencia nacional del Ministerio de Salud.
